# Performance Characterization of Radar-Based Delamination Assessment in Glass Fiber Reinforced Composites

**DOI:** 10.3390/s26113510

**Published:** 2026-06-02

**Authors:** Manuel E. Rao, Vittorio Memmolo, Jochen Moll, Peter Kraemer

**Affiliations:** 1 Department of Mechanical Engineering, University of Siegen, Paul-Bonatz-Straße 9-11, 57076 Siegen, Germany; jochen.moll@uni-siegen.de (J.M.); peter.kraemer@uni-siegen.de (P.K.); 2 Department of Industrial Engineering, Università degli Studi di Napoli Federico II, Via Claudio 21, 80125 Naples, Italy; vittorio.memmolo@unina.it

**Keywords:** structural health monitoring, probability of detection, receiver operating characteristic, damage detection, millimeter-waves, FMCW radar, composite structures

## Abstract

Radar technology in the microwave and millimeter-wave frequency range is the subject of current research for structural health monitoring of composite materials, e.g., damage detection in wind turbine blades. Performance assessment, enabling widespread practical application of this promising and non-contact sensing approach, can be realized via probability of detection (POD) theory, which is a statistical method for determining the detectability of damage through response metrics as a function of flaw size. This paper deals with the experimental investigation of a delamination model represented by two parallel glass fiber reinforced polymer plates separated from each other from 0mm to 1mm in steps of 0.01mm. Experimental studies with a frequency modulated continuous wave radar are performed under laboratory conditions in the frequency range from 57GHz to 65GHz. The signal response is represented by two damage indicators (DIs), according to the root mean square deviation and Mahalanobis distance. Since the reflection of electromagnetic waves exhibits a nonlinear behavior, this also implies a nonlinear response in the DI characteristic. The novelties in this work are the successful implementation of a nonlinear regression model, combined with an optimal threshold decision through receiver operating characteristic curves for a high-resolution POD representation. The POD with 95% confidence bounds indicates the flaw size at which the delamination can be detected reliably. Depending on the radar distance in experimental studies, the binary structural condition (damaged or undamaged) was correctly assessed from 95% to 100%. The minimum detectable size ranges from 0.01mm to 0.08mm.

## 1. Introduction

According to Mueller et al. [1], the approach for performance assessment of a structural health monitoring (SHM) system can be described in pyramidal form with four main contribution factors:

(1) The structure itself as the monitoring object;
(2) The requirements definition, including the level of SHM (e.g., detection or localization) as well as the environmental and operational conditions (EOCs) in which the structure is used;
(3) The setup with the sensing approach, including data analytics;
(4) The probability of detection (POD) assessment and evaluation of the receiver operating characteristic (ROC).

Each level is a prerequisite for reaching the next level. The POD is the final level, in which the quality, capability, reliability, and applicability of an SHM system are estimated.

As known from conventional non-destructive testing (NDT), POD curves indicate the size of any type of damage, also known as the flaw size, which can be detected with a given probability [2]. The use of the POD in various NDT methods was first published in 1974 by Rummel et al. [3]. Since then, further approaches have been developed, adapted, and expanded for diverse applications [4].

There are different POD methods, and the choice may depend on the specific application. They are listed and explained in Table 1 based on the review by Tai et al. [5]. In binary hit/miss analysis, either damage (hit) or no damage (miss) can be detected when damage is present [4]. The $\hat{a}$ versus *a* representation uses a signal response function for the POD assessment. A linear, semi-logarithmic, or double-logarithmic regression model is often applied to represent the signal response. Adjustments are necessary, depending on the NDT method, the scattering of the measurement data, and time-dependent correlations, as well as environmental and operational changes [2].

There are four common functions for transforming the POD into a generalized linear model depending on the flaw size: log-odds (logit), log-normal (probit), complementary loglog (cloglog) and loglog [6]. Typically, a POD of 90% with a 95% confidence level is required for damage detection [4]. For SHM systems that enable damage localization, the probability of localization can be determined within a tolerance radius [2].

In ROCs, the POD is plotted versus the probability of false alarms (PFAs). Hits and misses are therefore directly compared in order to qualify an SHM-capable system [4]. An optimal classifier for damage classification, e.g., a threshold value, is characterized by maximizing the POD and minimizing the PFAs [7]. Maes et al. [8] used the definition of the Youden index (YI) to evaluate the binary damage classification of a railway bridge in the context of vibration-based SHM. This metric is also used in this work for optimal decision making.

Composite materials have an increasing impact in many applications, because they are lightweight, cost-effective, and have a high stiffness [9]. Studies on the POD relating to SHM of carbon fiber reinforced polymer (CFRP) structures have been conducted, in particular, using ultrasonic methods. Tschöke et al. [10] investigated a CFRP plate with piezo sensors and tested the feasibility of a model-assisted POD approach. A special case of a CFRP structure is the omega stringer for airframes, which was investigated by Mueller et al. [1] in a climatic chamber with applied artificial damage. For the POD estimation of delamination sizes in CFRP, Kim et al. [6] used ultrasound imaging and Falcetelli et al. [11] optical fiber sensors.

An interesting approach is predictive POD, applied by Orellana et al. [12] to estimate the POD of two setups using analytical models without considering damaged states: SHM of a polyamide cuboid using contact ultrasonic testing and SHM of a CFRP plate using air-coupled ultrasonic testing. The work of Jiang et al. [13] presents a logistic regression model for the visual inspection of low-velocity impact damage on laminates, whose performance is evaluated using ROC curves and compared with that of machine learning architectures. The baseline-free identification and localization of delamination in a simulated glass fiber reinforced polymer (GFRP) plate is proposed by Jagadeeshwar et al. [14] in order to reduce the sensor density evaluated through ROCs.

Broadband microwave and millimeter-wave radar systems are useful for contact and non-contact inspections of composite structures [15]. Their first application in the SHM field as interferometers in the 1990s was aimed at detecting structural changes in civil infrastructure, e.g., a traffic bridge, the Leaning Tower of Pisa, and a wind turbine [16]. SHM of wind turbine blades (WTBs) is of interest in current research. They generally consist of GFRP. Due to the transparency of microwaves through GFRP, structural defects can be detected inside the composite layers [9].

**Figure 1:**
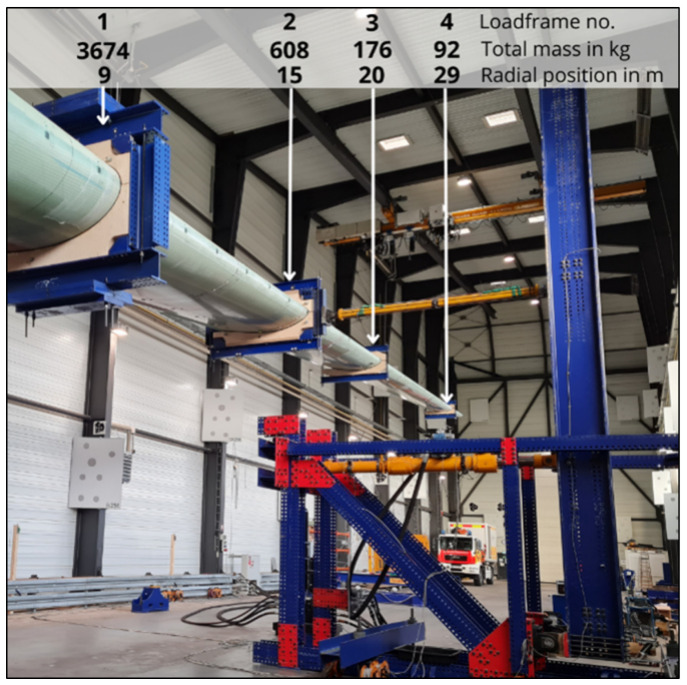
Radar-based SHM of a WTB at Fraunhofer IWES during a full-scale fatigue test. The WTB is excited in segmental vibrations in the horizontal plane by means of four load frames. The FMCW radars are embedded in several locations of the WTB. Taken from Simon et al. [17].

Embedded frequency modulated continuous wave (FMCW) radars showed promising results during a full-scale fatigue test of a 31m long WTB performed by Simon et al. [17]. The WTB in the test hall at Fraunhofer IWES is shown in Figure 1. In a further study by Streser et al. [18], the measurement data was used to train, validate, and test a convolutional neural network. Rao et al. [19] performed measurements with a FMCW radar on a GFRP sandwich with a modeled delamination. Based on the extracted physical properties, a damage model consisting of solid rigid foam with a thickness of 1.64mm and erosion protection tape was developed. This damage model was applied to a WTB section and detected with a FMCW radar mounted on the main web [9].

Delamination thicknesses are typically specified in fractions of millimeters. Mandell and Cairns [20] designed a skin-stiffener specimen for WTB fatigue loading in order to plot a displacement curve for delamination produced under different loads. The final separation of orthophthalic polyester 63-AX-051 was achieved at an actuator displacement of approximately 1.75mm. Li et al. [21] investigated delamination on a finite element model of a WTB spar cap with depths of 1.74mm, 5.22mm, and 8.7mm. Fang et al. [22] performed measurements with a vector network analyzer on GFRP plates with thicknesses of 0.75mm and 1.5mm. The delamination thicknesses were 0.03mm, 0.05mm, 0.1mm, and 0.2mm.

In CFRP, Wallentine et al. [23] monitored unidirectional CFRP matrix composite laminate plates using ultrasonic testing and serial sectioning microscopy. The delamination thickness was verified in a micrograph to be less than 0.05mm. Li et al. [24] created a finite element model of a CFRP structure in order to simulate delamination due to buckling with depths of 0.1mm, 0.5mm, and 1mm. The detection of hollow and material-filled holes in E-glass epoxy composites with thicknesses between 0.5mm and 5.5mm was investigated by Gokul et al. [25] with a vector network analyzer.

Wind turbines have rotor blades ranging in length from 20m to 100m. WTBs with greater lengths can generate more energy due to their larger frontal area for incident wind. However, this also increases the load levels, which leads to greater damage sizes. The literature refers to the lateral dimensions in this context [26]. In the full-scale fatigue test by Simon et al. [17] mentioned above, a 1.5m long crack spans almost completely the lateral length from the trailing edge to the leading edge. In the full-scale fatigue test by Al-Khudairi et al. [27] of a 47.5m long WTB, the length of an induced crack along the web was 1 m, and the length of the delamination was 25mm. Samareh-Mousavi et al. [28] investigated fatigue delamination growth in a 31m long WTB. The total delaminated area in the spar cap increased to $750{\mathrm{cm}}^{2}$. Desmond et al. [29] tested actuator displacements on two 8.3m long WTBs with different loads. The first WTB, consisting of a fiberglass spar cap, reached a displacement range of 0.59m. The second WTB, consisting of a carbon fiber spar cap, reached a displacement range up to 0.67m.

Apart from radar systems, the POD is plotted as a function of flaw size in other electromagnetic (EM) approaches. Pulsed thermography was used by Liu et al. [30] for the detection of artificial flat-bottom holes in a CFRP specimen. In the numerical simulation performed by Bao et al. [31], a coil installed over a conductive plate was detected using eddy current. Guided EM waves were coupled into a long pipe by Chen et al. [32] to characterize pipe wall thinning. Xu et al. [33] analyzed in their work the POD as a function of the signal-to-clutter ratio for weak target detection. Moreover, Memmolo et al. [34] studied omega stringer debondings through a microwave leakage approach.

The main contribution of this work is the successful implementation of a nonlinear POD approach for the performance assessment of a radar-based SHM methodology. Damage indicators (DIs) are calculated from experimental data using a FMCW radar at 60GHz, according to the root mean square deviation (RMSD) and Mahalanobis distance (MD) method. Numerical simulations are performed to compare DI trends with experimentally determined DIs.

The numerical model and experimental setup are both a sandwich of two rectangular GFRP plates, which are shifted from each other from 0mm to 1mm in 0.01mm steps to simulate typical delamination thicknesses over the entire plates. Based on a specified threshold, POD curves are calculated with 95% confidence bounds in order to obtain statistical information about the minimum detectability of a delamination using the DI approaches. To optimize the threshold, the maximum YI is taken into account, which is determined from the respective ROC curve.

The following aspects describe the novelty of this article:

- POD assessment of a radar-based SHM technique for delamination detection in composite structures using an idealized delamination model for GFRP plates;
- Comparison of different nonlinear regression models for reproducing the DI trends with a finer step width of the flaw size;
- Presentation and discussion of different methods for optimal threshold decision and their practical applicability;
- Physical explanation of the POD curves for minimal delamination detection, according to the slope, horizontal shift and distance to 95% confidence bounds in the context of electromagnetic testing;
- Outlook for future investigations with the delamination model on more complex composite structures.

The remainder of this paper is structured as follows: Section 2 provides an introduction to the mathematical formalism of the nonlinear POD, taking into account the ROC curves, as well as the numerical model and the experimental setup. Section 3 discusses the experimental POD results in relation to the accuracy of binary damage classification with optimally selected thresholds and 95% confidence bounds. Finally, Section 4 provides a summary of this work and an outlook for future research.

## 2. Materials and Methods

### 2.1. Damage Indicator Approaches for Radar Signals

FMCW radars continuously transmit EM waves within a specified bandwidth through an antenna. A proportion of the EM waves is reflected back from a target to the radar or transmitted through the target. The reflected signal is mixed with the input signal transmitted without and with a phase shift of 90° by the radar and discretized with $N_{\mathrm{pts}}$ measurement points. It is common to measure several frequency ramps of the same structural state due to statistical fluctuations. The total number of measurements is denoted by $N_{\mathrm{meas}}$. In summary, the complex radar signal in the time domain is expressed by $S\in C^{N_{\mathrm{pts}}\times N_{\mathrm{meas}}}$.

In SHM, signals of damaged states are mathematically combined with reference signals of the intact structure for comparison. The calculation of DIs is one way of determining structural differences and enabling damage identification. Let

(1) $$\Delta S_{mn}=S_{\mathrm{dam},n}\left[t_{m}\right]-{\bar{S}}_{\mathrm{ref}}\left[t_{m}\right]$$

be the difference between the signals of an arbitrary signal $S_{\mathrm{dam}}$ and the mean of all baseline signals ${\bar{S}}_{\mathrm{ref}}$ (super-reference) related to any point in time $t_{m}$. According to the RMSD method [9], the DIs are calculated as follows:

(2) $${\mathrm{DI}}_{\mathrm{RMSD},n}=\sqrt{\frac{1}{N_{\mathrm{pts}}}\sum_{m=0}^{N_{\mathrm{pts}}-1}{\left|\Delta S_{mn}\right|}^{2}}.$$

Here, the phase information is taken into account in the differences in the real and imaginary parts of the signals. For the MD method [35], the distances are calculated for the real and imaginary parts separately:

(3) $${\mathrm{DI}}_{\mathrm{MD},n}=\sqrt{\frac{1}{N_{\mathrm{pts}}}\sum_{m=0}^{N_{\mathrm{pts}}-1}{\left|{\mathrm{MD}}_{mn}^{\prime }+i{\mathrm{MD}}_{mn}^{\prime \prime }\right|}^{2}},$$

where *i* is the complex number, and ${\mathrm{MD}}_{mn}^{\prime }$ and ${\mathrm{MD}}_{mn}^{\prime \prime }$ are the Mahalanobis distance matrices for the real and imaginary part of the signal differences $\Delta s_{n}$ per measurement *n*:

(4) $${\mathrm{MD}}_{mn}=\mathrm{diag}\left\{\sqrt{{\left(\Delta s_{n}\right)}^{T}\Sigma^{-1}\left(\Delta s_{n}\right)}\right\}.$$

Σ denotes the covariance matrix of $S_{\mathrm{dam}}$ and ${\bar{S}}_{\mathrm{ref}}$. Only the diagonal elements are included, because they represent the variances at the same time $t_{m}$. Both methods use differential signals as input and should therefore result in similar DI trends.

### 2.2. Hit/Miss Analysis

Depending on a selected threshold, binary hit/miss statistics can be performed to determine whether present damage was detected or not. All DIs above the threshold are classified as damaged. Consequently, all DIs below the threshold value are classified as undamaged. Table 2 shows the four different possibilities for binary damage classification in tabular form. In the literature, this representation is referred to as a confusion matrix. For an accuracy of 100%, all hits and misses must lie on the main diagonal. The secondary diagonal indicates that the detection does not correspond to the true label.

Based on Table 2, various metrics are introduced [36]: the true positive rate (TPR), recall or sensitivity

(5) $$\mathrm{TPR}=\frac{\mathrm{TP}}{\mathrm{TP}+\mathrm{FN}},$$

the false positive rate (FPR) or the complement of specificity

(6) $$\mathrm{FPR}=\frac{\mathrm{FP}}{\mathrm{TN}+\mathrm{FP}},$$

and the accuracy of classification

(7) $$\mathrm{accuracy}=\frac{\mathrm{TN}+\mathrm{TP}}{N}.$$

The plot of the TPR as a function of the FPR is known as the ROC. The YI is defined by the difference between the TPR and FPR in the interval $\left[0,1\right]$:

(8) YI=TPR−FPR.

The larger the YI, the better the threshold was chosen [8].

However, a maximum YI means that the FPs are minimal due to the difference in (8), but FNs are still present. Depending on the application, it may also be advantageous to minimize the FNs in order to rule out the possibility of an already damaged condition being detected too late or not at all. In this case, the YI can be redefined to TPR−FNR, where the FNR is the false negative rate $\frac{\mathrm{FN}}{\mathrm{FN}+\mathrm{TP}}$. The maximum of this difference minimizes the FNs. Two other ways to find the optimal threshold would be via $\min\left\{\mathrm{FP}+\mathrm{FN}\right\}$ or via the standard deviation of the reference DIs. For real-time applications, the threshold must be calibrated before starting the measurement series.

### 2.3. Probability of Detection in $\hat{a}$ Versus *a* Representation

The hit/miss analysis aims to assess the POD by counting the TPs and FNs. In $\hat{a}$ versus *a* representation, the POD is calculated more in a statistical way and plotted continuously as a function of the flaw size *a*. Let

(9) $$\hat{a}=f\left(a\right)$$

be the estimated signal response through nonlinear regression of DIs of damaged structural states, where *f* denotes the fit function. The 95% confidence bounds are calculated using the Delta method (specified in MIL-HDBK-1823A [37]). The standard deviation of the estimated signal response is given by

(10) $$\sigma_{\mathrm{DI}}=\sqrt{\frac{1}{N_{\mathrm{meas}}-1}\sum_{n=1}^{N_{\mathrm{meas}}-1}{\left({\mathrm{DI}}_{n}-{\hat{a}}_{n}\right)}^{2}}.$$

Here, $\hat{a}$ is vectorized equally to $\mathrm{DI}\in R^{N_{\mathrm{meas}}}$ with $N_{\mathrm{states}}$ different values. $N_{\mathrm{states}}$ denotes the number of structural states.

Once the fit function *f* has been defined, $\hat{a}\left(a\right)$ can be extended with $N_{\mathrm{reg}}$ regression points for better resolution, i.e., $\hat{a}\in R^{N_{\mathrm{meas}}}\to \hat{a}\in R^{N_{\mathrm{reg}}}$. The scattering of the estimated function $\hat{a}$ relative to the true DIs is calculated by the normal probability density function (PDF) to receive the Gaussian bell curves for each structural state [38]:

(11) $$\mathrm{PDF}\left(\frac{\mathrm{DI}-\hat{a}}{\sigma_{\mathrm{DI}}}\right)=\frac{1}{\sqrt{2\pi }\sigma_{\mathrm{DI}}}\;e^{-\frac{{\left(\mathrm{DI}-\hat{a}\right)}^{2}}{2\sigma_{\mathrm{DI}}^{2}}}.$$

The POD covers all detected and present damages in case the DIs are above the selected threshold. Since the definition of the normal cumulative distribution function (CDF) is used to calculate the POD, the complement has to be taken into account in order to change the relation symbol as follows [2]:

(12) $$\begin{array}{cc} \mathrm{POD}=\mathrm{Pr}\left(\hat{a}>{\hat{a}}_{\mathrm{th}}\right) & =1-\mathrm{Pr}\left(\hat{a}\leq {\hat{a}}_{\mathrm{th}}\right)\\ \; & =1-\Phi \left(\frac{{\hat{a}}_{\mathrm{th}}-\hat{a}}{\sigma_{\mathrm{DI}}}\right), \end{array}$$

where Φ is the discrete normal CDF [38]:

(13) $$\Phi \left(\frac{{\hat{a}}_{\mathrm{th}}-\hat{a}}{\sigma_{\mathrm{DI}}}\right)=\frac{1}{\sqrt{2\pi }\sigma_{\mathrm{DI}}}\sum_{k=-\infty }^{{\hat{a}}_{\mathrm{th}}}e^{-\frac{{\left(k-\hat{a}\right)}^{2}}{2\sigma_{\mathrm{DI}}^{2}}}.$$

For a finite number of points, −∞ should be interpreted as the starting point. The functional relationship in (12) refers to the “probit” representation of the POD [6].

A POD of 50% represents only random processes [7]. A POD of 90% assumes the minimum detectability of damage. Two characteristic parameters are crucial here: $a_{90}$ specifies the flaw size at a POD of 90%. On the other hand, $a_{90/95}$ specifies the flaw size at a POD of 90% with the lower 95% confidence bound. With a small $\sigma_{\mathrm{DI}}$, the POD with and without confidence bounds converge, ensuring greater reliability in damage identification.

### 2.4. Experimental Setup

**Figure 2:**
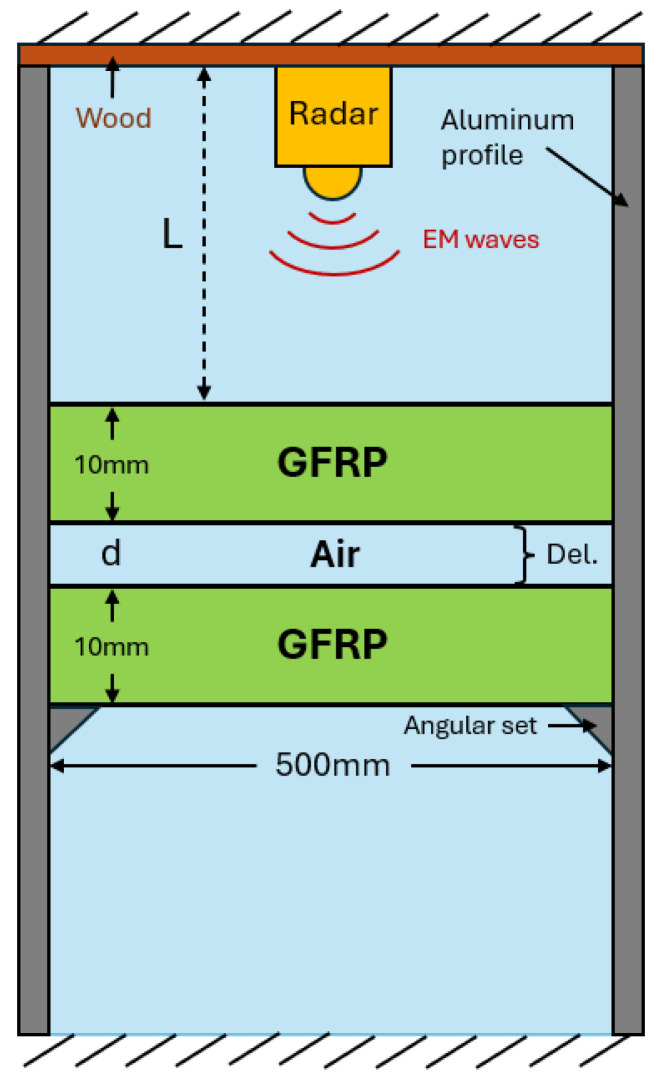
Schematic visualization for the conceptual idea of a represented delamination in a GFRP structure, according to Rao et al. [19], reproduced courtesy of The Electromagnetics Academy.

The experimental setup in the laboratory consists of a sandwich of two rectangular GFRP plates from CG TEC GmbH (Spalt, Germany) of dimensions 500mm×500mm×10mm, with air as the intermediate layer to simulate a delamination extended over the entire plates. The flaw sizes are the delamination thicknesses *d* from 0mm to 1mm increased in steps of 0.01mm so that a total of 101 structural conditions were measured. Figure 2 demonstrates the principal idea to construct a numerical model and experimental setup.

**Figure 3:**
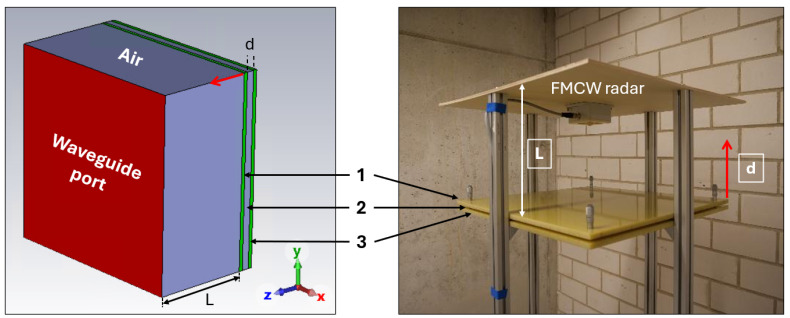
Visualization of the numerical model (**left**) and experimental setup (**right**) of a represented delamination. *L* denotes the radar distance to the first GFRP plate (1), and *d* denotes the delamination thickness directed towards the radar (red arrows). Label (2) is the intermediate layer consisting of air, and (3) is the second GFRP plate, according to Rao et al. [19], reproduced courtesy of The Electromagnetics Academy.

Numerical simulations are performed in CST Microwave Studio (2024 version) only to verify the equivalent trend in the signal response in the form of DIs. For this purpose, a graphics processing unit consisting of an AMD Ryzen 3950X processor from Advanced Micro Devices, Inc. (Santa Clara, CA, USA) with 16 cores, 128GB of random-access memory and a 32GB Nvidia Quadro GV100 graphics card from Nvidia Corporation (Santa Clara, CA, USA) was used. The numerical model in CST Microwave Studio and the experimental setup in the laboratory are shown in Figure 3.

The signal source in the simulation is a waveguide port for the excitation of plane waves with the time domain solver, while in the experiments, the sR60-12RLi radar from IMST GmbH (Kamp-Lintfort, Germany) is placed centrally on the GFRP plates on a wooden plate. In both cases, EM waves with a bandwidth of 8GHz from 57GHz to 65GHz are excited. In the experiment, the different delamination thicknesses *d* are set by separating the upper GFRP plate parallel to the lower GFRP plate using micrometer screw gauges of type 148-104-10 from Mitutoyo (Neuss, Germany), with a nonius scale accuracy of 0.01mm. Furthermore, the lower plate is fixed to aluminum profiles with angular sets from item Industrietechnik GmbH (Solangen, Germany). The wooden plate aligns the 1.3m long aluminum profiles perpendicular to the optical table to increase mechanical stability and to prevent friction when moving the upper GFRP plate.

Preliminary studies [19] have shown the appearance of minima in the DI trends represented by the RMSD method, each half of the wavelength 2.46mm related to the center frequency of 61GHz. This is explained by the physical knowledge of resonances that occur within the delamination layer. In the case of resonance, the electromagnetic fields disappear at the edges, so that no phase shifts have to be considered during reflection and transmission at the interface. Another finding was the approximate independence of DIs from various distances *L* between the signal source and the first interface.

**Figure 4:**
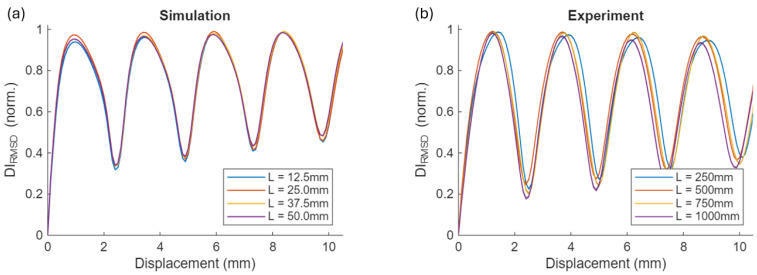
(**a**) Numerical and (**b**) experimental RMSD trends as a function of the displacement of two GFRP plates directed towards the radar in four different radar distances *L*. The damage size step width is 0.1mm, according to Rao et al. [19], reproduced courtesy of The Electromagnetics Academy.

For verification purposes, the RMSD trend is shown in Figure 4 for delamination thicknesses from 0mm to 10.5mm in steps of 0.1mm. In order to assign a single delamination thickness to each DI value, only DIs up to 1mm are used for the POD assessment, with a finer step width of 0.01mm, which corresponds to the tolerance of the micrometer screw gauges. Because the simulation represents only an idealistic delamination model without any noise in the signals or changes in the mechanical properties, only one *L* is considered for the comparison of numerically and experimentally determined DI trends. Due to limited computing capacity, the simulation involves a 20-fold reduction in dimension except for the delamination thickness *d*, because this parameter is crucial for the differences in the signal response. The distance from the waveguide port to the first interface is fixed at 12.5mm.

However, in the experiment, both GFRP plates are fixed at four points so that bending is unavoidable. This leads to unevenness, which complicates the scattering behavior of electromagnetic waves and affects the SHM of GFRP plates that are shifted relative to each other. The reason lies in the greater surface coverage of the radar as *L* increases, which can be proved by trigonometric derivations. Since each experiment involves different reference datasets, four different radar distances *L* were investigated in the experimental studies: 250mm, 500mm, 750mm, and 1000mm.

**Figure 5:**
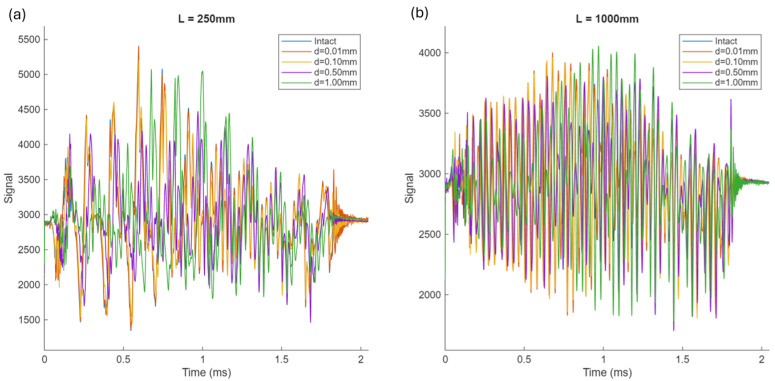
Averaged signals in the time domain for (**a**) L=250mm (**b**) and 1000mm, with four different delamination thicknesses *d* for comparability. The signals differ with increasing *d* from the reference of the intact structure.

Reference signals are only applied to signals from any structure in the same measurement series with the same radar distance *L*, since signals in the time domain consist of more periods with a higher *L*, as can be seen exemplarily in Figure 5 for L=250mm and L=1000mm. Here, 20 ramps are averaged. It can be figured out that the differences in the reference signal increase with higher delamination thickness *d* until 1mm. A time gate in the interval of $\left[0.20,1.78\right]\mathrm{ms}$ is applied because of random noise at the beginning and the end of the signal, caused by the analog digital converter of the radar system.

The dielectric lens of the radar focuses the radiation of EM waves at 8° in azimuth and 7° in elevation so that only a small area can be monitored more precisely with higher signal intensity. The number of data points per radar measurement is $N_{\mathrm{pts}}=2048$. With $N_{\mathrm{ramps}}=20$ frequency ramps per structural state and $N_{\mathrm{states}}=101$ structural states, this results in a total of $N_{\mathrm{meas}}=2020$ measurements, where 20 measurements are recorded with the intact structure and 2000 with a delamination. More information about the radar system can be found in Table 3.

## 3. Results and Discussion

**Figure 6:**
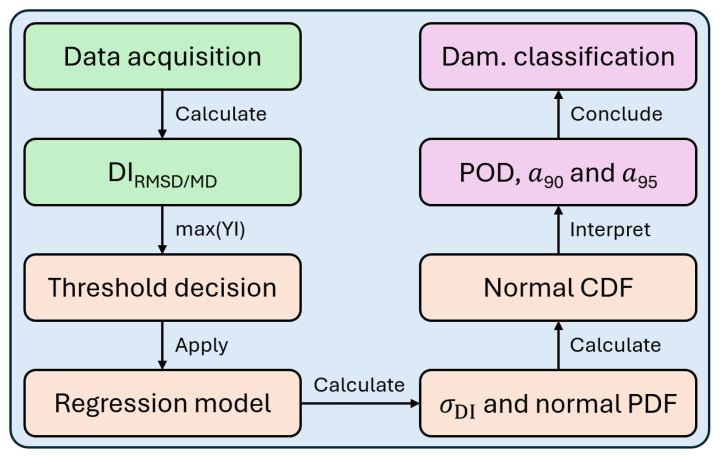
Flow chart from data acquisition and processing (light green boxes) to calculation (light orange boxes) and interpretation of the POD (light purple boxes), from which the delamination thickness of the damaged state can be reliably detected.

The procedure for calculating and interpreting the POD curves is shown in Figure 6. First, simulation data for one waveguide port distance and experimental data with four different radar distances *L* are recorded. Afterwards, DIs are calculated after the RMSD and MD to receive the signal responses of the signals in the time domain. To find the optimal threshold ${\hat{a}}_{\mathrm{th}}$ for each measurement series, ROC curves are plotted, and the YIs are calculated. The maximum YI indicates the best threshold. After applying a suitable fit function, the standard deviation of the signal response $\sigma_{\mathrm{DI}}$ and the normal PDF per structural state are calculated from the DIs.

Using ${\hat{a}}_{\mathrm{th}}$, $\sigma_{\mathrm{DI}}$ and the regression points $\hat{a}$, the POD is obtained with the parameters $a_{90}$ and $a_{90/95}$. These parameters indicate a detected delamination with a 90% POD and an additional 95% confidence. Finally, the predicted structural conditions are compared with the true label to determine the accuracy of the damage identification using the proposed DI approach.

### 3.1. Representation of the Signal Response Through Damage Indicators

**Figure 7:**
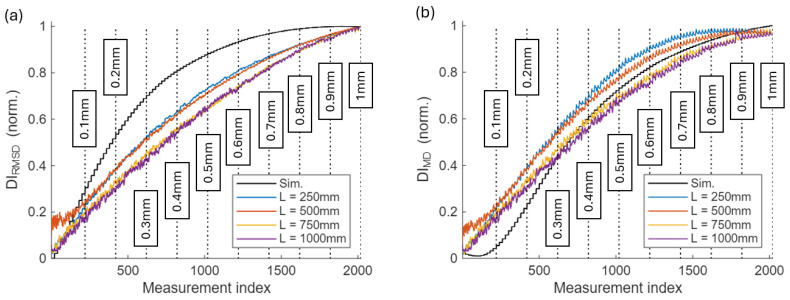
Numerically and experimentally calculated normalized (**a**) ${\mathrm{DI}}_{\mathrm{RMSD}}$ and (**b**) ${\mathrm{DI}}_{\mathrm{RMSD}}$ values for four different *L* and $d=\left[0\mathrm{mm},1\mathrm{mm}\right]$ in steps of 0.01mm. Each structural state is measured using 20 frequency ramps. In total, 2020 measurements were performed. The simulation corresponds to L=12.5mm.

The DIs are calculated according to Equations (1) and (4) and are plotted in Figure 7 for the simulation and experimental data as a function of the delamination thickness *d*. The DIs of a structural state are shown within one bar. Overall, the DI trends are similar between the simulation, experiment and the applied DI methods, and increase from the undamaged state to 1mm delamination thickness. Plateaus for a small *d* can be recognized in the simulation due to the absence of statistical deviations and random noise per measurement. In addition, lower modes propagate in this one-dimensional problem due to the much smaller geometry that leads to different slopes. For comparability, all DIs are normalized to one.

In the undamaged case, the experimentally determined DIs fluctuate more, suggesting that the plates were not completely parallel to each other in reality. In particular, for L=500mm, the ${\mathrm{DI}}_{\mathrm{RMSD}}$ values of the reference state and damaged states until d=0.07mm overlap, meaning that this state cannot be clearly assigned to a damaged state. Therefore higher thresholds must be chosen for the POD assessment. When comparing Figure 7a,b, the MD is more robust to these fluctuations.

### 3.2. Threshold Decision

**Figure 8:**
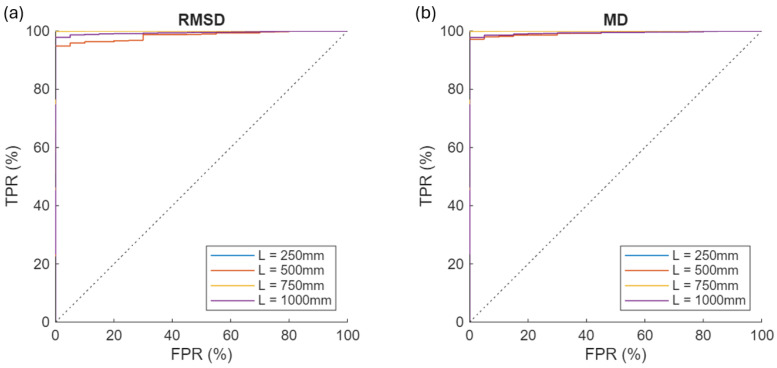
Experimentally determined ROC curves for the (**a**) RMSD and (**b**) MD method. The blue line that is almost completely overlapped with the yellow line represents a perfect damage classification, while the yellow, purple and especially the red line show some misclassifications. For comparison, the dotted black line theoretically represents only random processes.

The number of regression points is set to $N_{\mathrm{reg}}$ = 100,000. Since the normalized DIs range within $\left[0,1\right]$, the threshold ${\hat{a}}_{\mathrm{th}}$ is increased in steps of $N_{\mathrm{reg}}^{-1}$ within this interval. The DIs are classified as shown in Table 2. The TPR and FPR are calculated according to Equations (5) and (6) in order to plot the ROC curves. These are shown in Figure 8 for the RMSD and MD method separately. An intersection with the diagonal represents only random processes and is referred to as a POD of 50%. A perfect classification is given by a constant TPR value of 100%.

**Figure 9:**
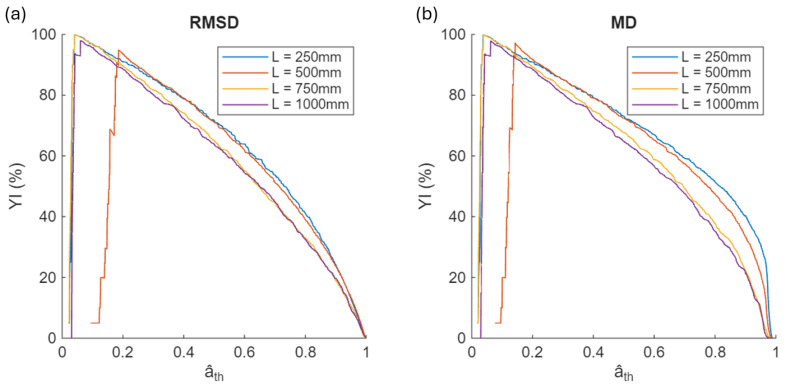
Experimentally determined YIs for the (**a**) RMSD and (**b**) MD method, calculated from the ROC curves of Figure 8. The higher the YI, the better the threshold for the minimization of misclassification.

Afterwards, the YIs are calculated using Equation (8). This formulation was used due to the small amount of DIs of reference (20) compared to the amount for damaged states (2000). The YIs are plotted in Figure 9 as a function of ${\hat{a}}_{\mathrm{th}}$. The optimal threshold that is used for calculating the POD is derived from the maximum YI.

For L=250mm, YI=1 occurs for one ${\hat{a}}_{\mathrm{th}}$, which indicates perfect classification. For the measurement series with L=750mm, the YI trends look quite similar for both DI methods. Compared to L=500mm and L=1000mm, more misclassifications that are recognized in the respective ROC curves lead to a smaller maximum of the YI. In addition, a higher threshold has to be selected. L=500mm produces the worst damage classification results. The physical reasons are discussed later in Section 3.4.

Since the same number of ramps was measured for all structural states, the ratio between intact and damaged structures is highly unbalanced. To counteract this, difference signals relative to a super-baseline were used according to Equation (1), and the baseline signals were considered as noise in the POD assessment. To determine only positive predictions for unbalanced datasets in more detail, precision–recall analysis is often performed. The positive predictive value (PPV) or precision is defined by [36]

(14) $$\mathrm{PPV}=\frac{\mathrm{TP}}{\mathrm{TP}+\mathrm{FP}}.$$

**Figure 10:**
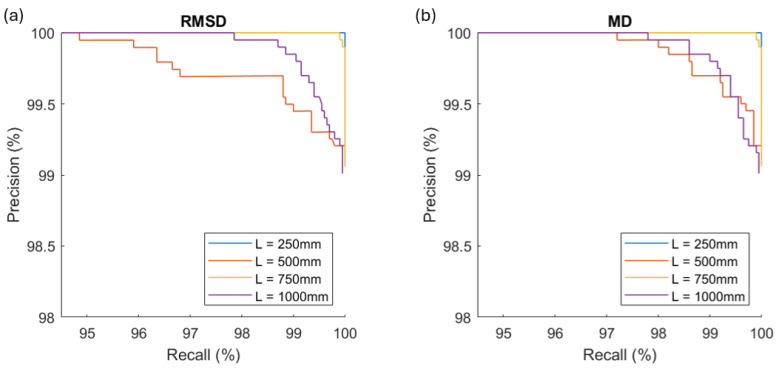
Precision–recall analysis for the (**a**) RMSD and (**b**) MD method. The best classification results for positive prediction is given with a recall and precision of 100% constantly.

In Figure 10, the curves for all radar distances and DI methods are shown. In particular, for the RMSD and measurement series with L=500mm, it is striking that a higher recall strongly decreases the precision due to the increase in FPs.

### 3.3. Regression Model

The regression model uses a fit function that covers the measurement points as continuously as possible. In addition to the fit curve, upper and lower 95% confidence bounds represent the acceptance range of a two-sided Gaussian test, which correspond to approximately ±1.96 times the standard deviation [38]. A well-chosen fit function is characterized by upper and lower confidence bounds being as close as possible to the fit curve.

**Figure 11:**
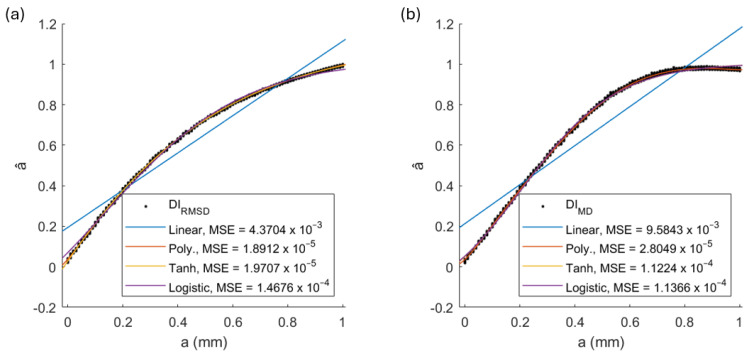
Regression models for experimentally determined (**a**) ${\mathrm{DI}}_{\mathrm{RMSD}}$ and (**b**) ${\mathrm{DI}}_{\mathrm{MD}}$ values with L=250mm. For each structural state, 20 frequency ramps are measured.

Looking at the DI trends in Figure 7a,b, it becomes apparent that the trend is nonlinear and closely resembles a saturation function. Four regression models were tested empirically and compared via the mean squared error (MSE)

(15) $$\mathrm{MSE}=\sum_{n}{\left({\hat{a}}_{n}-{\mathrm{DI}}_{n}\right)}^{2}$$

for suitability: a linear, ninth order polynomial, hyperbolic tangent and logistic regression function. Figure 11 plots the different regression lines in a joint graph for comparison. It has been found that the polynomial function is the most suitable fit with the smallest MSE. Due to the low interpretability and the choice of a saturation function, the hyperbolic tangent is selected according to the following equation:

(16) $$f\left(a\right)=c_{0}+c_{1}\tanh\left(c_{2}\;a+c_{3}\right).$$

**Figure 12:**
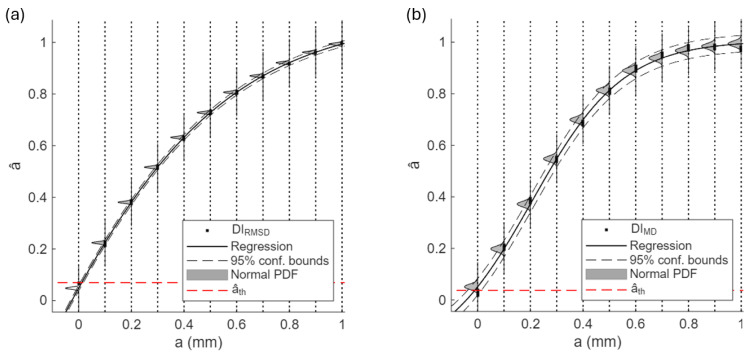
Nonlinear regression line with upper and lower 95% confidence bounds and normal distribution lines of experimentally determined (**a**) ${\mathrm{DI}}_{\mathrm{RMSD}}$ and (**b**) ${\mathrm{DI}}_{\mathrm{MD}}$ values with L=250mm. For each structural state, 20 frequency ramps are measured. The red dashed line represents the threshold decision.

The result of the nonlinear regression with 95% confidence bounds is shown in Figure 12 for ${\mathrm{DI}}_{\mathrm{RMSD}}$ and ${\mathrm{DI}}_{\mathrm{MD}}$ values with a radar distance of L=250mm. In addition, the normal PDF per structural state, calculated according to Equation (11), and the optimal threshold are also plotted. For viewing purposes, DIs for eleven structural states are plotted, and the Gaussian bell curves are normalized to 0.05. Only the width of the distribution is important in order to estimate the scattering of the measurement points. The Gaussian bell curves are broader for the DIs calculated after the MD method. This means that the regression function is more suitable for the RMSD method. Since the threshold ${\hat{a}}_{\mathrm{th}}$ is below all DIs of the damaged states, zero FNs are counted.

### 3.4. Probability of Detection Assessment

**Figure 13:**
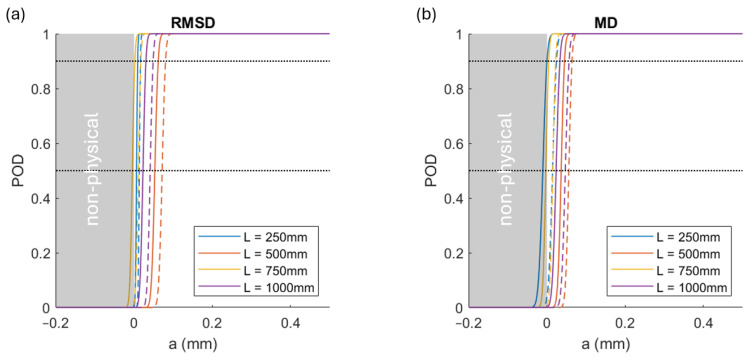
Experimentally determined POD curves for (**a**) ${\mathrm{DI}}_{\mathrm{RMSD}}$ and (**b**) ${\mathrm{DI}}_{\mathrm{MD}}$ trends. The solid and dashed lines represent the POD and POD with lower 95% confidence bounds respectively. Intercept points with the dotted black lines represent the minimum detectability of a delamination at a POD level of 90% and random processes at a POD level of 50% at a certain thickness.

The experimental POD curves with lower 95% confidence bounds are plotted in a joint graph in Figure 13. They are calculated using Equations (10)–(13). The mathematical formulation of the regression model gives negative values for the flaw size *a*, which are non-physical. The area below a=0mm is shaded gray in the POD graphs. The first physical value is a=0mm for the intact condition. The POD levels of 50% for random processes and 90% for the minimum detectability of damage are plotted as well. If the intersections of the POD curves with these levels are below the physical limit, this means that there are no random processes, and damage detection is possible from the first damaged state.

Two aspects need to be discussed in order to assess the quality of the measurements:

- The slope of the respective POD curve and the position of the 95% confidence bounds depend on the suitability of the regression model.
- The shift along the horizontal axis depends on the suitability of the threshold and measured flaw sizes.

The simulation does not show any scattering of the DIs within a structural state and is therefore excluded in this section. Nevertheless, the threshold can be set close to ${\hat{a}}_{\mathrm{th}}=0\mathrm{mm}$. This enables unambiguous classification, and the POD curve resembles a step function.

The regression model seems to be a good approximation for both DI methods and all measurement series. Therefore, the lower 95% confidence bound does not differ significantly from the fit curves. It can be figured out that the RMSD and MD methods result in similar POD curves. Looking at Figure 13a, the POD curve for L=500mm is shifted along the horizontal axis, since the structural state d=0.07mm is still classified as undamaged with the selected threshold. The first state classified as damage is d=0.08mm. The POD curve for the DIs determined with L=1000mm points out some misclassifications compared to smaller radar distances L=250mm and 750mm.

The comparison of the relevant parameters ${\hat{a}}_{\mathrm{th}}$, $a_{90}$ and $a_{90/95}$ for the experimental POD curves can be found in Table 4 and Table 5. The results of the damage classification with accuracy determined according to Equation (7) are listed in Table 6 and Table 7. The overall accuracy ranges from 94.90% to 100.00% and the minimal detectable delamination thickness from 0.01mm to 0.08mm. The increase in $a_{90}$ for the largest radar distance to the first interface L=1000mm is striking. To avoid reflections at aluminum profiles and unevenness of the GFRP plates, among other things, a stronger focusing with the radar becomes important.

For both the RMSD and MD method, L=250mm shows perfect accuracy to distinguish between the intact and damaged structure due to the presence of delamination in the GFRP sandwich model. The accuracies below 100% for the other POD curves are explained by the overlap of DIs of damaged states with the intact structure. Strong fluctuations in the reference signal for L=500mm lead to higher overlaps, which degrade damage detection at an early stage.

One reason for the large fluctuations, in particular the DIs of the intact structure, is the suboptimal calibration of the zero point when the micrometer screw gauges are reset for the next measurement series. The four-point mounting on the aluminum profiles results in partial unevenness of the GFRP plates. Therefore, the setup needs to be optimized to improve reproducibility, e.g., by using precise step motors. Another reason for the increase in FNs with a larger *L* lies in trigonometric considerations. With a larger *L* and a constant radiation pattern of the radar, the covered area on the GFRP plate increases. Unless the plate is completely flat, greater random scattering affects the detectability of the delamination model.

Interestingly, the classification results for L=250mm and L=750mm are equal for both DI methods, for L=1000mm, they are similar, and for L=500mm, they are different. Discrepancies between the methods may appear due to different assumptions. The similarity between RMSD and MD is the use of a difference signal, but the fundamental difference and advantage of the MD method is the use of variances, which dampen strong fluctuations in the reference signal for L=500mm.

The strength of the RMSD is in the simple implementation, short computation time, and intuitive interpretability through successive differentiation from a baseline signal. However, assigning equal weight to all signals, associated with neglecting noise, has a disadvantageous effect on SHM. For varying EOCs, this can lead to misclassification of damage. The MD is used in the literature as a distance measure for identifying outliers in multivariate statistics under variable EOCs [35]. For statistically fluctuating signals that correlate with other signals of the same structural state, the MD is superior to the RMSD due to the use of a covariance matrix. Statistical stability is thus given greater weight, which also increases the gain by increasing the number of measurements. However, this can be a disadvantage in real-time applications, as it increases the computational load.

This laboratory study has limitations when the problem is addressed in real-world scenarios. The primary focus is on validating an idealized damage model for the qualification of SHM systems without damaging the composite structure itself. Full-scale fatigue tests or loaded specimens in a more controlled environment are two options for characterizing real damage. As soon as signal changes caused by EOCs play a role, a more in-depth analysis is necessary. Alipek et al. [39] demonstrate the application of GuidedGradCAM, which is an explainable machine learning technique for classifying local changes in radar reflections caused by wind speed, rotational speed, pitch angle, or nacelle orientation in radar images. The main task in that work was ensuring robust differentiation in all three rotor blades of a wind turbine using a tower radar.

## 4. Conclusions

This work deals with the radar-based identification of a simulated delamination with thicknesses from 0mm to 1mm in a sandwich model with GFRP. Data was obtained experimentally with a FMCW radar under laboratory conditions and numerically in CST Microwave Studio at frequencies ranging from 57GHz to 65GHz. DIs were calculated in the time domain using the RMSD and MD method. The simulations were only performed to verify the similarities of DI trends. The POD framework was used to evaluate the detectability of a delamination with the proposed DI algorithms.

The innovation was the combination of a nonlinear regression function and optimal thresholding methods. The DI trends exhibited a periodic, nonlinear behavior. For the POD analysis, the DI trend up to the first maximum was taken into account to unambiguously assign the DIs to a delamination thickness. Within this local interval, the trend could be approximated by a saturation function. Moreover, the hyperbolic tangent produced fit results with a low MSE to calculate high-resolution POD curves for experimentally acquired datasets. The most suitable method for optimal thresholding was determining the maximum of the YI, since data was recorded for each structural state with the same number of ramps under laboratory conditions.

With a POD of 90% and additional lower 95% confidence bounds, the minimum detectability of damage is given in the $\hat{a}$ versus *a* representation. Different slopes, standard deviations, and shifts on the horizontal axis were obtained for the normal CDFs. Detectability became more difficult with increasing radar distances from delamination thicknesses 0.01mm to 0.08mm. Potential reasons are the unevenness of the GFRP plates, mechanical inaccuracies of the experimental setup and statistical fluctuations in the radar measurements. The accuracy of the binary damage classification in the hit/miss analysis ranges from 95% to 100%.

Based on these laboratory studies and findings, the delamination model will be investigated in a climate chamber under variable but controllable temperature and humidity settings in future research. Since the radar signals are affected by changes in the permittivity of the materials or by changes in the resistance of the electronics, the implementation of compensation methods is essential.

Furthermore, a field study on a wind turbine is planned, which involves a realistic variation in EOCs. A wide radar network will be installed inside two rotor blades to acquire data on the intact structure, with a lightweight version of the delamination model. Since wind turbine operating companies prohibit damaging the structure itself for the qualification of SHM systems, the conception, testing and evaluation of the damage model is crucial. The functional dependence of radar signals on numerous time-varying environmental and operational parameters is a highly complex issue and requires, in many cases, the use of machine learning to classify structural states from EOCs.

## Figures and Tables

**Table 1:** Description of various POD methods, according to [5].

POD Method	Explanation
Binary hit/miss	This analysis counts hits and misses in the presence of damage.
$\hat{a}$ versus *a*	The signal response $\hat{a}$ is a function of the flaw size *a*, represented by a suitable regression model.
Bayesian approaches	Including prior knowledge and probability distributions to characterize uncertainties, Bayesian statistics can estimate posterior POD trends.
Monte Carlo simulation	Monte Carlo simulations generate random data samples with realistic measurement conditions and flaw sizes. In a model-assisted approach, the POD can be accurately estimated.
29/29 method	For all 29 flaw sizes, the POD should be equal to 100%.
Maximum likelihood estimation	This approach identifies the parameters of a probability distribution based on measured data to reach the highest likelihood function.
Rayleigh–Rice method	The POD is estimated considering background noise (Rayleigh) and incorrect signals (Rice) separately.

**Table 2:** Four possible classifications of the estimated structural state relative to the true state [2].

Damage	Absence	Presence
Not detected	True negative (TN)	False negative (FN)
Detected	False positive (FP)	True positive (TP)

**Table 3:** Specification of the sR60-12RLi radar module from IMST GmbH, which is used in FMCW mode for the experiments, taken from the data sheet.

Parameter	Specification
Max. frequency range	57GHz up to 65GHz
Antenna type	Chip-integrated patch antenna
Channels	1 Tx and 2 Rx
Antenna characteristics	65° azimuth × 60° elevation
Antenna characteristics & dielectric lens	8° azimuth × 7° elevation
Effective radiated power	−15dBm up to 13dBm
Effective radiated power & dielectric lens	−3dBm up to 25dBm
Antenna polarization	Linear
Max. number of samples	2048
Sampling frequency	1MHz
Operating power	2W
Power supply	10.5V up to 40V
Temperature	−40 °C up to 60 °C
Dimension	110mm × 84mm × 52mm
Weight	200g

**Table 4:** POD results for experimentally determined ${\mathrm{DI}}_{\mathrm{RMSD}}$ values, given by ${\hat{a}}_{\mathrm{th}}$, $a_{90}$ and $a_{90/95}$.

*L*	${\hat{a}}_{\mathrm{th}}$	$a_{90}$ (mm)	$a_{90/95}$ (mm)
250mm	0.041	0.009	0.016
500mm	0.186	0.062	0.080
750mm	0.040	0.002	0.017
1000mm	0.060	0.030	0.049

**Table 5:** POD results for experimentally determined ${\mathrm{DI}}_{\mathrm{MD}}$ values, given by ${\hat{a}}_{\mathrm{th}}$, $a_{90}$ and $a_{90/95}$.

*L*	${\hat{a}}_{\mathrm{th}}$	$a_{90}$ (mm)	$a_{90/95}$ (mm)
250mm	0.038	0.000	0.024
500mm	0.143	0.045	0.064
750mm	0.039	0.004	0.022
1000mm	0.062	0.033	0.056

**Table 6:** Binary damage classification results for experimentally determined ${\mathrm{DI}}_{\mathrm{RMSD}}$ values.

*L*	TN	FP	TP	FN	Accuracy $\left(\%\right)$
250mm	20	0	2000	0	100.00
500mm	20	0	1897	103	94.90
750mm	20	0	1998	2	99.90
1000mm	20	0	1957	43	97.87

**Table 7:** Binary damage classification results for experimentally determined ${\mathrm{DI}}_{\mathrm{MD}}$ values.

*L*	TN	FP	TP	FN	Accuracy $\left(\%\right)$
250mm	20	0	2000	0	100.00
500mm	20	0	1944	56	97.23
750mm	20	0	1998	2	99.90
1000mm	20	0	1956	44	97.82

## Data Availability

The datasets presented in this article are not immediately available because of further research purposes. Requests to access the datasets should be directed to the corresponding author of this manuscript.

## Abbreviations

The following abbreviations are used in this manuscript:

POD	Probability of detection
DI	Damage indicator
SHM	Structural health monitoring
FMCW	Frequency modulated continuous wave
ROC	Receiver operating characteristic
EOC	Environmental and operational condition
NDT	Non-destructive testing
PFAs	Probability of false alarms
YI	Youden index
CFRP	Carbon fiber reinforced polymer
GFRP	Glass fiber reinforced polymer
WTB	Wind turbine blade
EM	Electromagnetic
RMSD	Root mean square deviation
MD	Mahalanobis distance
TN	True negative
FN	False negative
FP	False positive
TP	True positive
TPR	True positive rate
FPR	False positive rate
PDF	Probability density function
CDF	Cumulative distribution function
MSE	Mean squared error
